# Ice-Houses

**Published:** 1871-12

**Authors:** 


### ICE-HOUSES.

Ice is an important remedy in various forms of disease, and is of great value in families as a means of keeping food and fruits fresh, nutritious, and healthful; hence every well-to-do family, owning its own homestead, or two or three neighboring families, would do well to make an arrangement for an abundant supply of this summer luxury, as it may be done at small cost, by building an ice-house on the "Stevens Plan." For one family, make a house twelve feet each way, by setting twelve posts in the ground, three on a side; board it up, eight feet high, on the inside, so that the weight of the ice shall not press the boards outward; dig out the dirt inside, six inches deep, and lay down twelve inches of sawdust; pack the ice in a pile nine feet each way, filling the space of eighteen inches between the ice and the boards with sawdust or tan bark, with the same thickness on top; make an old-fashioned board roof, leaving the space above the ice open for ventilation. Have a small entrance on the north side of the roof.

If the ice-house can be located on the north side of a hill, and a small stream of water introduced slowly through the roof, on a very cold day, so as to make its way between the pieces of ice, the whole mass would freeze solid; or a pile of snow could thus be made into solid ice, and would last from one winter to another.

